# Gas Sensing Mechanism and Adsorption Properties of C_2_H_4_ and CO Molecules on the Ag_3_–HfSe_2_ Monolayer: A First-Principle Study

**DOI:** 10.3389/fchem.2022.911170

**Published:** 2022-05-12

**Authors:** Lufen Jia, Jianxing Chen, Xiaosen Cui, Zhongchang Wang, Wen Zeng, Qu Zhou

**Affiliations:** ^1^ College of Engineering and Technology, Southwest University, Chongqing, China; ^2^ Department of Quantum and Energy Materials, International Iberian Nanotechnology Laboratory (INL), Braga, Portugal; ^3^ School of Materials and Energy, Southwest University, Chongqing, China; ^4^ College of Materials Science and Engineering, Chongqing University, Chongqing, China

**Keywords:** the first-principle study, HfSe_2_ monolayer, Ag_3_ doping, gas adsorption, transformer oil

## Abstract

The detection of dissolved gases in oil is an important method for the analysis of transformer fault diagnosis. In this article, the potential-doped structure of the Ag_3_ cluster on the HfSe_2_ monolayer and adsorption behavior of CO and C_2_H_4_ upon Ag_3_–HfSe_2_ were studied theoretically. Herein, the binding energy, adsorption energy, band structure, density of state (DOS), partial density of state (PDOS), Mulliken charge analysis, and frontier molecular orbital were investigated. The results showed that the adsorption effect on C_2_H_4_ is stronger than that on CO. The electrical sensitivity and anti-interference were studied based on the bandgap and adsorption energy of gases. In particular, there is an increase of 55.49% in the electrical sensitivity of C_2_H_4_ after the adsorption. Compared to the adsorption energy of different gases, it was found that only the adsorption of the C_2_H_4_ system is chemisorption, while that of the others is physisorption. It illustrates the great anti-interference in the detection of C_2_H_4_. Therefore, the study explored the potential of HfSe_2_-modified materials for sensing and detecting CO and C_2_H_4_ to estimate the working state of power transformers.

## Introduction

Two-dimensional transition metal dichalcogenides (TMDs) have recently emerged as a focus of the scientific community in virtue of their versatile and tunable physical properties (Xu et al., 2013; Duerloo et al., 2014; Si et al., 2020; Ju et al., 2021). Layered transition metal disulfides (LTMDs) own the properties of large specific surface value, high electronic activity, and sensitivity (Choi and Kim, 2018). The properties mentioned previously contribute to the great potential of the chemical sensors. Hafnium dichalcogenides (HfX_2_, X = S, Se, or Te) belong to middle-gap semiconductors and have a high chemical reactivity and various energy dispersions (Yue et al., 2015; Mirabelli et al., 2016; Mleczko et al., 2017; Cruzado et al., 2021). As a consequence, all the excellent properties mentioned previously embody their future applications in optoelectronics and electronics.

As the most important and valuable piece of equipment, the operation status of the oil-immersed power transformers affects the safety and stability of the electric power system operation directly (Gui et al., 2020). The malfunction that occurred in oil or the insulating paper is inevitable during the long-running process of the transformer. The malfunction mainly includes overheating of oil or the insulating paper, arc discharge, partial discharge, and spark discharge (Tang et al., 2020). Under the effect of electricity and heat, the transformer oil will undergo a chemical reaction and generate gases. The gases generated are mostly hydrocarbons and some other related gases, such as CH_4_, C_2_H_4_, CO_2_, and CO. Their composition and contents are closely linked to the type and severity of the transformer faults (Singh and Bandyopadhyay, 2010). Dissolved gas analysis (DGA) could discover the hidden faults sensitively. DGA mainly includes infrared spectroscopy, gas chromatography, Raman spectroscopy, and the gas sensor method (Tran et al., 2018). Due to its simple structure, high reaction sensitivity, low cost, and low power consumption, the gas sensor method is proposed to be applied for detecting gases by more and more scientists (Wei et al., 2020).

HfSe_2_ has the atomic stacking structure with a layer of Hf atoms stuck in the middle of two layers of Se atoms. Cui et al. explored the adsorption behavior of Pd-doped and Pt-doped HfSe_2_ monolayers upon several kinds of gases, such as NO_2_, SO_2_, and SOF_2_ (Cui et al., 2020a; Cui et al., 2020c). It is found that the Pd-doped monolayer behaves better for NO_2_, while the Pt-doped HfSe_2_ monolayer behaves better for SO_2_. Wang et al. studied the adsorption nature and behavior of Rh-doped HfSe_2_, and it shows a stronger interaction between the SO_2_ molecule and the monolayer than SO_2_F_2_ (Wang and Liao, 2019). Wu et al. studied the doping behavior of Pd atoms and the sensing character of the Pd-doped HfSe_2_ (Pd–HfSe_2_) monolayer upon CO and C_2_H_2_ (Wu et al., 2022). They found that the Pd-HfSe_2_ monolayer possesses a better adsorption behavior on CO. Yang et al. investigated the adsorption of CO, C_2_H_2_, and C_2_H_4_ based on the Cu-doped Se-vacant MoSe_2_ (Cu–MoSe_2_) monolayer (Yang et al., 2020). But Cu–MoSe_2_ is not selective for the detection of C_2_H_4_. Xu et al. explored four characteristic dissolved gases in transformer oil: H_2_, CH_4_, C_2_H_2_, and C_2_H_4_ (Xu et al., 2020). It was found that the adsorption effects on C_2_H_2_ and C_2_H_4_ are stronger than those of the others. Speaking of the methods of modifying the material monolayer, Asif et al. integrated positively charged semiconductive sheets of Zn-NiAl LDH and negatively charged layers of rGO to modify the glassy carbon electrode (Asif et al., 2019a). The modified electrode exhibited excellent electrocatalytic activity. The doping formation of the Ag_3_ cluster and the adsorption behavior of C_2_H_4_ and CO were not explored yet. In this article, the adsorption of C_2_H_4_ and CO of Ag_3_-doped HfSe_2_ monolayers was investigated. The doping formation adopted is the Ag_3_ cluster, and three atoms contribute to the formation of a stable triangle structure. The results showed that the Ag_3_-doped HfSe_2_ monolayer behaves better for C_2_H_4_ adsorption but is inactive for CO.

## Computational Details

The first-principle calculations based on the DFT (density functional theory) framework emerged as an important and dominant method in quantum mechanics simulation (Delley, 2000). All the calculations in this article adopted the DMol^3^ package in Materials Studio software to establish the adsorption model of pristine and Ag_3_-doped HfSe_2_ upon C_2_H_4_ and CO. Moreover, Perdew–Burke–Ernzerhof (PBE) functional with a generalized gradient approximation (GGA) was used for the electron exchange and correlation function (Chen et al., 2019b). To better deal with the van der Waals interactions and the relativistic effect of doped atoms, Tkatchenko and Scheffler’s (TS) method and the DFT semi-core pseudopotential (DSSP) method were employed, respectively. The atomic orbital basis set was described by the double numerical plus polarization (DNP) method (Cui et al., 2020b). The supercell geometry optimizations were calculated under the Monkhorst-Pack k-point mesh of 5 × 5 × 1, while 7 × 7 × 1 was sampled for the more accurate electronic structure calculations. To ensure the precision of total energy, smearing was set as 0.005Ha, and the energy tolerance accuracy, maximum force, and displacement were set as 10^−5^ Ha, 2 × 10^−3^ Ha/Å, and 5 × 10^−3^ Å, respectively (Topsakal et al., 2009; Sharma et al., 2018). To mimic a free-standing graphene sheet of HfSe_2_, a periodic 4 × 4 × 1 HfSe_2_ supercell was established with a 20-Å vacuum region imposed in the direction where the sheet is not periodic, and the vacuum region is large enough to ensure the doping and gas adsorption processes, as well as eliminate the interaction between adjacent units (Yang et al., 2020).

The binding energy $E_{b}$ , which plays an important role in estimating the most stable configuration, is calculated as follows.

$$E_{b}=E_{Ag_{3}-HfSe_{2}}-E_{Ag_{3}}-E_{HfSe_{2}}.$$ (2-1)

The aforementioned equation represents the total energy of the Ag_3_-HfSe_2_ monolayer subtracting the total energies of the free-standing sheet of the HfSe_2_ and Ag_3_ cluster. Comparing the binding energy of different doping sites, the configuration with the lowest binding energy is defined as the most stable one (Asif et al., 2022).

The adsorption energy $E_{ad}$ generated during the adsorption process is calculated as follows.

$$E_{ad}\;=\;E_{gas/Ag_{3}-HfSe_{2}}\;-\;E_{gas}\;-\;E_{Ag_{3}-HfSe_{2}}.$$ (2-2)

Here, in the aforementioned equation, $E_{gas/Ag_{3}-HfSe_{2}}$ represents the total energy of the system of the Ag_3_-doped HfSe_2_ monolayer with the adsorbed gas molecule. $E_{gas/Ag_{3}-HfSe_{2}}$ and $E_{Ag_{3}-HfSe_{2}}$ mean the energy of the adsorbed gas molecule and the energy of the Ag_3_-doped HfSe_2_ monolayer, respectively. By comparing the value of the adsorption energy $E_{ad}$ with 0.8, the type of the adsorption can be judged. If greater than 0.8, it is defined as chemisorption, otherwise, it is physisorption (Liu et al., 2021).

## Results and Discussion

### Analysis of Pristine and the Ag_3_-Doped HfSe_2_ Monolayer

**FIGURE 1:**
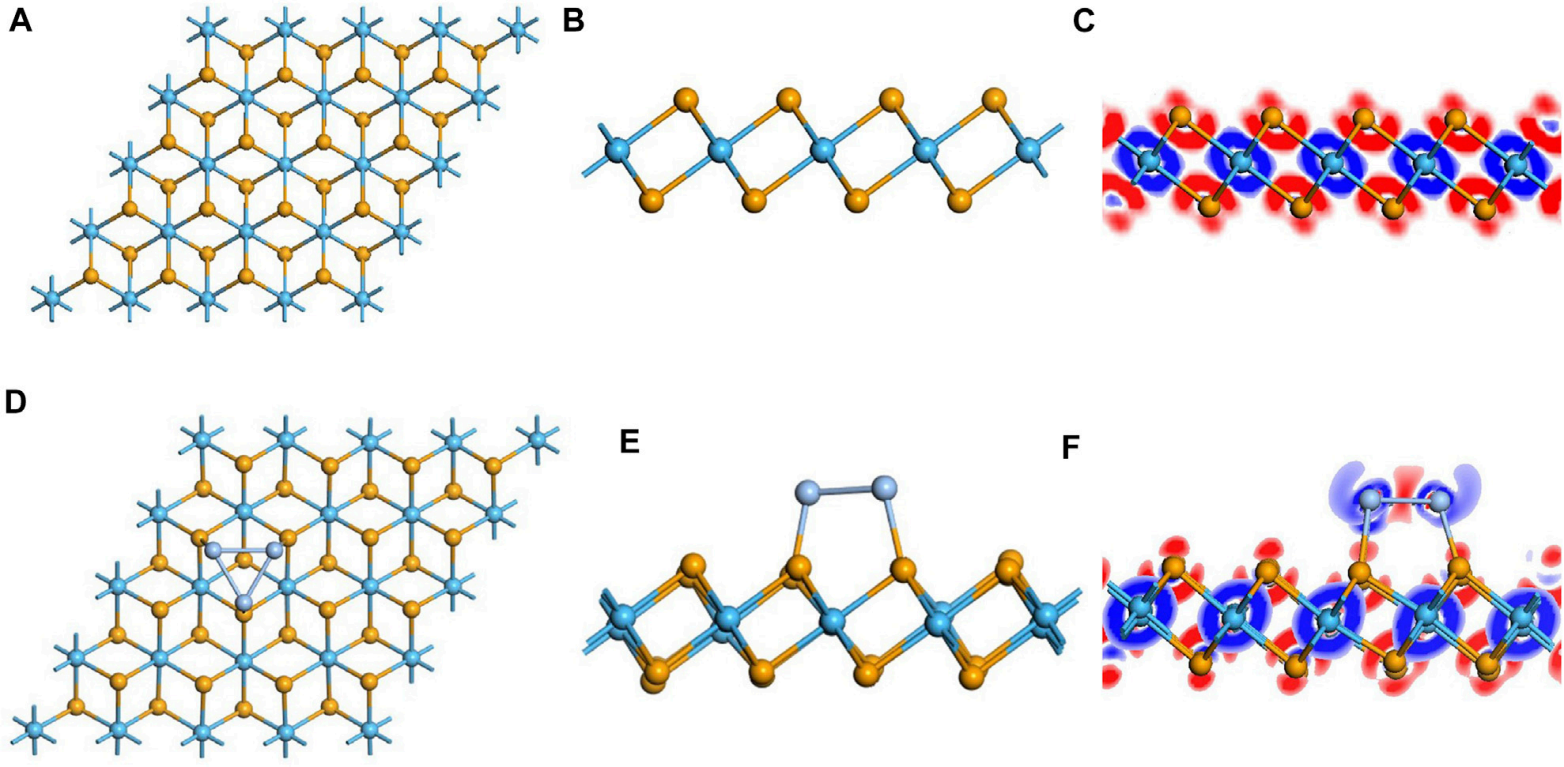
Optimized configuration and electronic structures of the pristine and doped monolayer. **(A–C)** pristine system and **(D–F)** doped system. In DCD, red and blue regions represent electron accumulation and deletion.

First, the geometric structure of the free-standing HfSe_2_ monolayer was optimized to obtain the most stable configuration, as shown in Figures 1A,B. It holds the atomic stacking structure with a layer of Hf atoms stuck in the middle of two layers of Se atoms (Wang and Liao, 2019). The Hf–Se bond in the intrinsic HfSe_2_ monolayer is measured as 2.70 Å, which is consistent with the articles published previously. Speaking of the performance of Ag_3_ doping on the HfSe_2_ monolayer, three doped sites were taken into account. They are named T_se1_ (a tripod site right upside the lower-layer Se atoms), T_se2_ (right upside the upper-layer Se atoms), and T_Hf_ (a tripod site right upside the mid-layer Hf atoms) (Ambrusi et al., 2017; Ju et al., 2017; Mi et al., 2021). As far as the most stable doped configuration, the lower the binding energy (*E*_b_), the more stable is the structure. *E*_b_ is calculated as shown in Eq. 2-1, and the lowest one was chosen as the most stable doping system in this article. The Ag_3_ cluster favored to be doped through the T_se1_ site with the numerical value of −2.512 eV, of which the *E*_b_ is lower than −1.347 and −1.155 eV for T_se2_ and T_Hf_, respectively.

**FIGURE 2:**
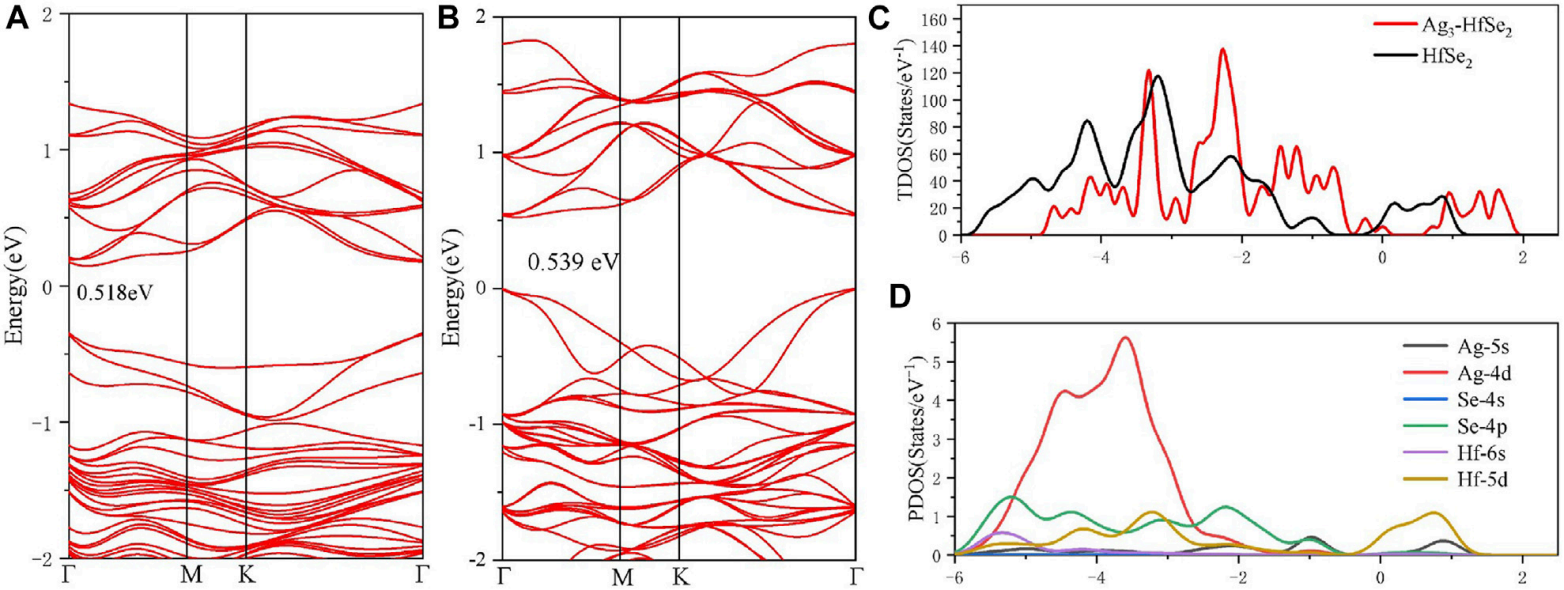
Band structure and the TDOS and PDOS of the pristine and doped monolayers. **(A)** Band structure of the pristine HfSe_2_ monolayer. **(B–D)** Band structure, TDOS, and PDOS of the Ag_3_–HfSe_2_ monolayer.

As is depicted in Figures 1D,E, there is a slight deformation after the doping of the Ag_3_ cluster in the HfSe_2_ monolayer, of which the Ag–Se bonds are 2.646, 2.628, and 2.648 Å, respectively. From the result of the Mulliken method (Chen et al., 2019a), it can be seen that the Ag_3_ cluster is positively charged by 0.267 e after doping. As is shown in Figure 1F, Ag atoms are surrounded by blue areas, in which the red and blue regions indicate electron accumulation and deletion independently (Cui et al., 2019). As a consequence, the analysis of the Mulliken method is in good accordance with the DCD, showing that the Ag_3_ cluster is an electron donator. Compared with the bandgap obtained as 0.539 eV of the pristine HfSe_2_ monolayer in Figure 2A, Ag_3_–HfSe_2_ is 0.553 eV with a decrease of 0.14 eV in Figure 2B. In comparison of the pristine and Ag_3_-doped HfSe_2_’s TDOS, it can be seen that there is a right shift, which is keeping with the result of the bandgap analysis.

As illustrated in Figure 2D, it can be seen from the PDOS that there are several overlapping regions among the Ag-4d, Se-4p, and Hf-5d orbitals at −6 ∼ −1 eV, coincidently between the Hf-5d and Ag-5s orbitals at 0–1.5 eV. The PDOS verifies the conclusion that there are strong interactions existing among Ag, Se, and Hf atoms (Hu et al., 2019; Gao et al., 2020). As a consequence, due to the doping performance of the Ag_3_ cluster, it is among the Ag-4d, Hf-5d, and Se-4p orbitals that the main orbital hybridization occurs. In addition, compared with the TDOS and PDOS mentioned previously, it can be seen that it is on the Ag, Se, and Hf atoms that the bottom of the conduction band and the top of the valence band are localized, and it also verifies the charge transfers from the Ag_3_ cluster to the Se and Hf atoms (Yang et al., 2019).

### Analysis of Gas Adsorption on the Pristine HfSe_2_ Monolayer

After investigating the doping performance of the Ag_3_ cluster, the adsorption behaviors of the pristine HfSe_2_ monolayer upon CO and C_2_H_4_ were investigated as follows. In terms of the adsorption site of different gases upon the pristine HfSe_2_ monolayer and Ag_3_–HfSe_2_ monolayer, all the possible adsorption sites were taken into account, but only the site with the lowest adsorption energy was adopted for further study (Zhang et al., 2009).

**FIGURE 3:**
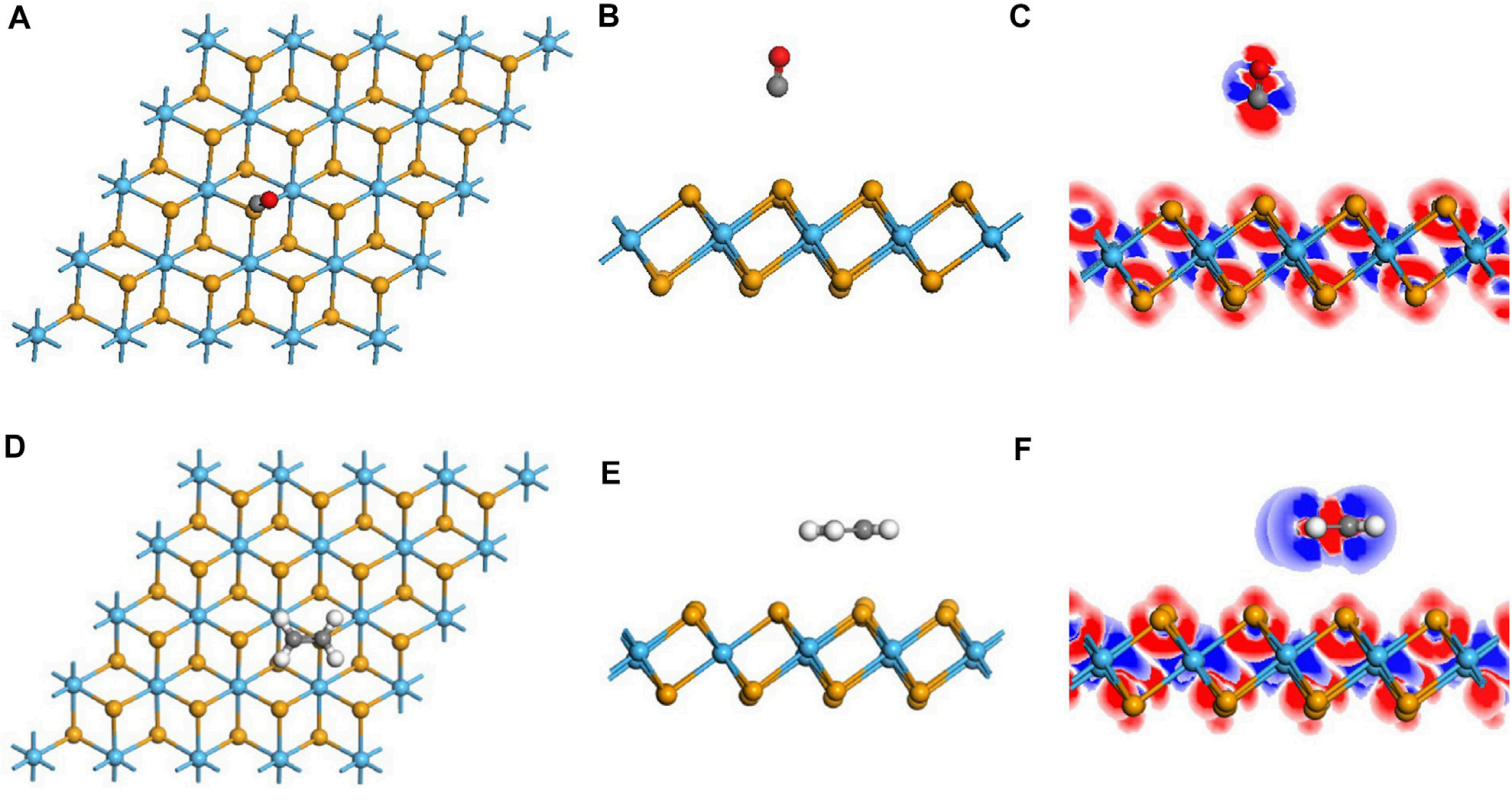
Geometric and electronic structures of the adsorption system of CO and C_2_H_4_ on the intrinsic monolayer. **(A–C)** Adsorption system of CO and related DCD. **(D–F)** Adsorption system of C_2_H_4_ and related DCD.

As for the adsorption of CO molecules on the pristine HfSe_2_ monolayer, three different adsorption directions were simulated. The molecule accessed the Se atoms *via* C and O atoms vertically and parallelly to the monolayer, respectively (Ma et al., 2016). As shown in Table 1, C atoms tend to be adsorbed by Se atoms in the upper layer of the pristine HfSe_2_ monolayer, with a distance of 4.055 Å between C and Se atoms. The length is significantly longer than the sum of the C and Se atoms’ covalent radii, verifying the inexistence of the C–Se bond (Zhou et al., 2018b). For the adsorption of CO on the pristine HfSe_2_ monolayer, the adsorption energy is 0.159 eV, which is less than 0.8 eV and to be defined as physisorption. The amount of charge transfer Q_T_ between the monolayer and CO molecule is very weak, with a value of 0.004 e. It includes 0.019 e of the C atom and −0.0105 e of the O atom (Late et al., 2014). Similarly, the DCD shown in Figure 3C reveals that the C atom and O atom are surrounded by blue and red areas, which is consistent with the Mulliken analysis. Furthermore, the bandgap, calculated as 0.691 eV, has an increase of 0.067 eV. As a consequence, based on the analysis mentioned previously, the adsorption effect of CO on the pristine monolayer is poor.

**TABLE1:** Adsorption characteristic parameters of CO and C_2_H_4_ on the pristine monolayer.

Adsorbed gas	Bond length (Å)		Mulliken charge (e)		Q(e)	E_ads_ (eV)	d(Å)
CO	C-O	1.141	C	0.108	0.004	−0.159	4.055
			O	−0.104			
C_2_H_4_	C_1_-C_2_	1.337	C_1_	−0.194	0.028	−0.344	3.389
	C_1_-H_1_	1.092	C_2_	−0.186			
	C_1_-H_2_	1.092	H_1_	0.102			
	C_2_-H_3_	1.092	H_2_	0.101			
	C_3_-H_4_	1.092	H_3_	0.101			
			H_4_	0.101			

For the adsorption sites on the pristine monolayer of C_2_H_4_, two possible sites were investigated to simulate, with C_2_H_4_ being vertical and parallel to the upper layer (Qian et al., 2020). According to the calculated adsorption energy, it is the site that in a parallel direction holds the lowest energy. The C_2_H_4_ molecule is arrested by the Se atom, with a distance of 3.39 Å between C and Se atoms, which is longer than the sum of their covalent radii (Gao et al., 2019). The charge transfer Q_T_ generated by the CO molecule is −0.015 e, including −0.378 e of C atoms and 0.363 e of H atoms, which is consistent with the DCD shown in Figure 3F. Compared with the monolayer before the adsorption, there is an increase of 0.067 eV for the bandgap. In terms of the adsorption energy, it is 0.3437 eV that reveals the property of physisorption, which is below the standard of 0.8 eV (Zhou et al., 2018a). By reason of the foregoing analysis, the adsorption capacity of the pristine monolayer for C_2_H_4_ is not very well either.

### Adsorption of the CO Molecule on the Ag_3_-Doped HfSe_2_ Monolayer

**FIGURE 4:**
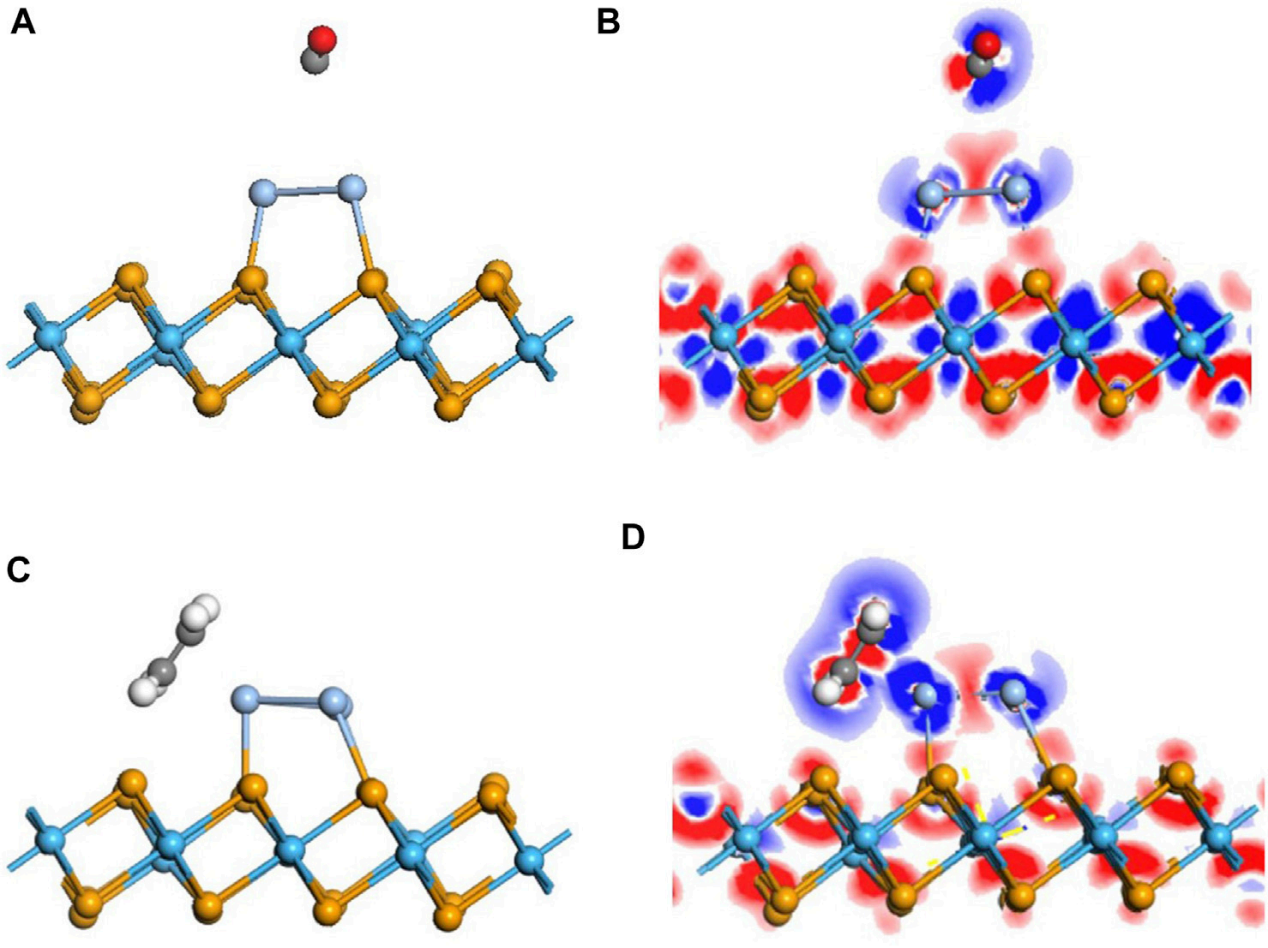
Geometric and electronic structures of the adsorption system of CO and C_2_H_4_ on the doped Ag_3_–HfSe_2_ monolayer. **(A,B)** Adsorption system of CO and related DCD. **(C,D)** Adsorption system of C_2_H_4_ and related DCD.

With regard to the adsorption of the doped monolayer, the adsorption of CO upon the Ag_3_-doped monolayer was investigated first. The most stable adsorption configuration is as same as the pristine one. As is depicted in Figure 4A, there is a slight deformation in the configuration after doping, manifesting the reduction of the distance between the C atom and dopant, elongated from that of 4.055 Å in the pristine system to 3.65 Å. But the distance measured as 3.65 Å is still similarly longer than the sum of the covalent radii, representing that there is no chemical effect (Cortés-Arriagada et al., 2018). Although E_ad_ is raised to 0.388 eV, its property is still defined as physisorption.

**FIGURE 5:**
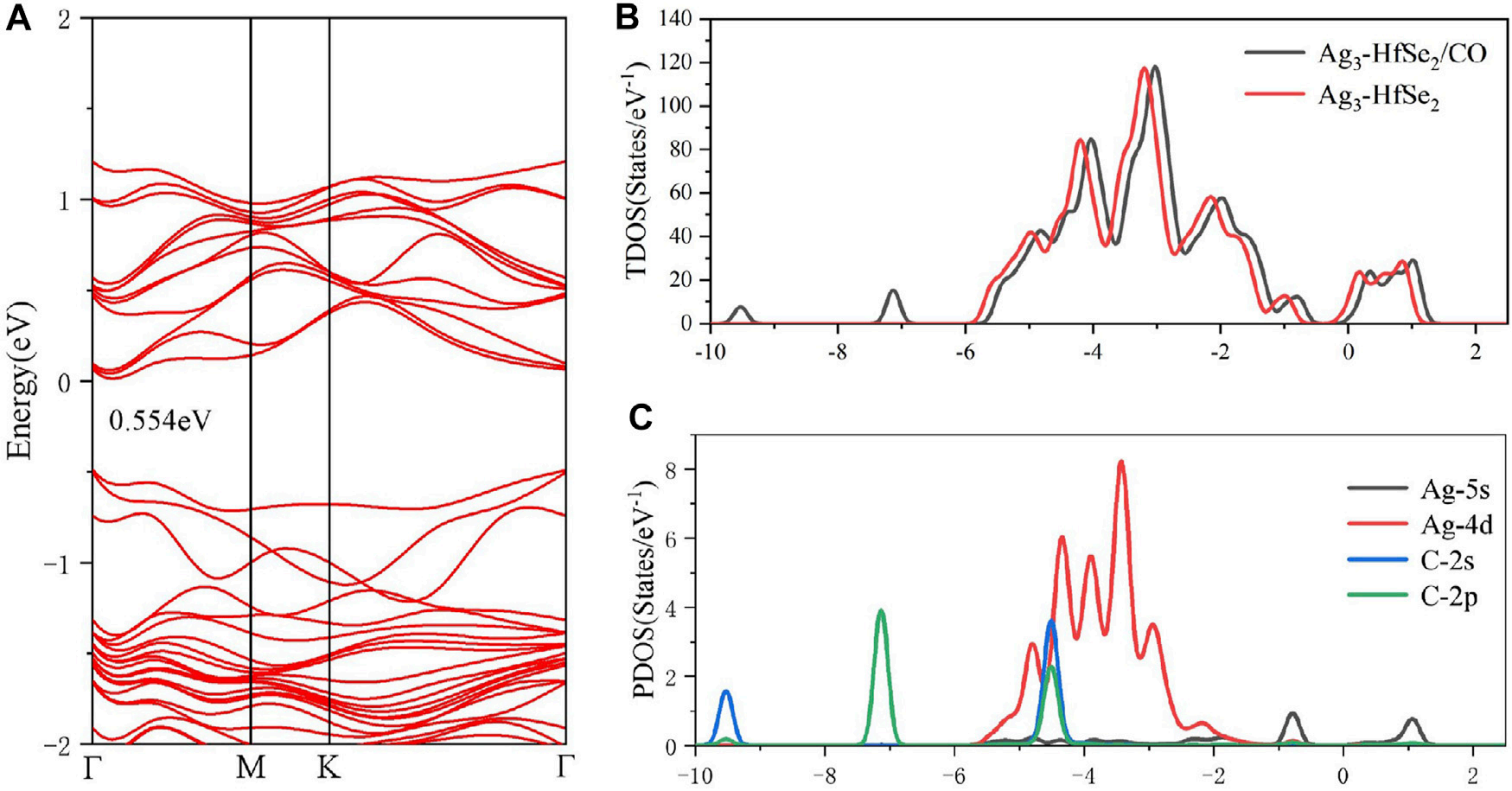
**(A)** Band structure, **(B)** TDOS and **(C)** PDOS of CO molecules’ adsorption on the Ag_3_-doped HfSe_2_ monolayer.

Figure 5B exhibits the total TDOS distribution of the adsorption system of CO, where a right shift is observed for the TDOS state of the CO adsorbed system after adsorption. By analyzing the band structure of the system after the adsorption of CO, it was found that the bandgap is 0.554 eV, which is shown in Figure 5A. Furthermore, as is shown in Table 2, there is 0.014 e transferred from the doped system to the CO molecule based on the analysis of Mulliken atomic charges (Allian et al., 2011). Combined with the Mulliken analysis, the demonstration of the DCD shown in Figure 5B is logical, in which the Ag atoms are surrounded by blue areas. In other words, the dopant Ag_3_ cluster plays an important role as an electron donator in the system. According to the PDOS in Figure 5C, it can be found that the Ag-4d orbital is highly hybrid with the C-2s and C-2p orbitals at −5 ∼ −4 eV, whereas the Ag-5s orbital is hybrid with the C-2p orbital at −1 ∼ −0.5 eV (Khan Musa et al., 2020). Also, after the adsorption of the CO molecule, the bandgap of the adsorbed system is raised to 0.554 eV, which has an increase of 0.015 eV.

**TABLE2:** Adsorption characteristic parameters of CO and C_2_H_4_ on the doped monolayer.

Adsorbed gas	Bond length (Å)		Mulliken charge (e)		Q(e)	E_ads_ (eV)	d(Å)
CO	C-O	1.143	C	0.0491	−0.031	−0.388	3.652
			O	−0.0801			
C_2_H_4_	C_1_-C_2_	1.367	C_1_	−0.191	0.221	−0.912	2.400
	C_1_-H_1_	1.092	C_2_	−0.188			
	C_1_-H_2_	1.092	H_1_	0.152			
	C_2_-H_3_	1.092	H_2_	0.162			
	C_3_-H_4_	1.091	H_3_	0.143			
			H_4_	0.143			

As a consequence, compared with the pristine system, there is a rise in the absolute value of the change in the bandgap. The change of the electronic parameters of the monolayer after the adsorption of CO molecules could be observed. Compared to the adsorption energy, adsorption distance, and charge transfer with those of pristine mentioned before, it can be seen that the adsorption performance of CO has been improved to a certain extent but is not ideal (Zhang et al., 2017). To draw a conclusion, it is not suitable for Ag_3_-doped HfSe_2_ either as a sensor material or as an adsorbent.

### Adsorption of the C_2_H_4_ Molecule on the Ag_3_-Doped HfSe_2_ Monolayer

**FIGURE 6:**
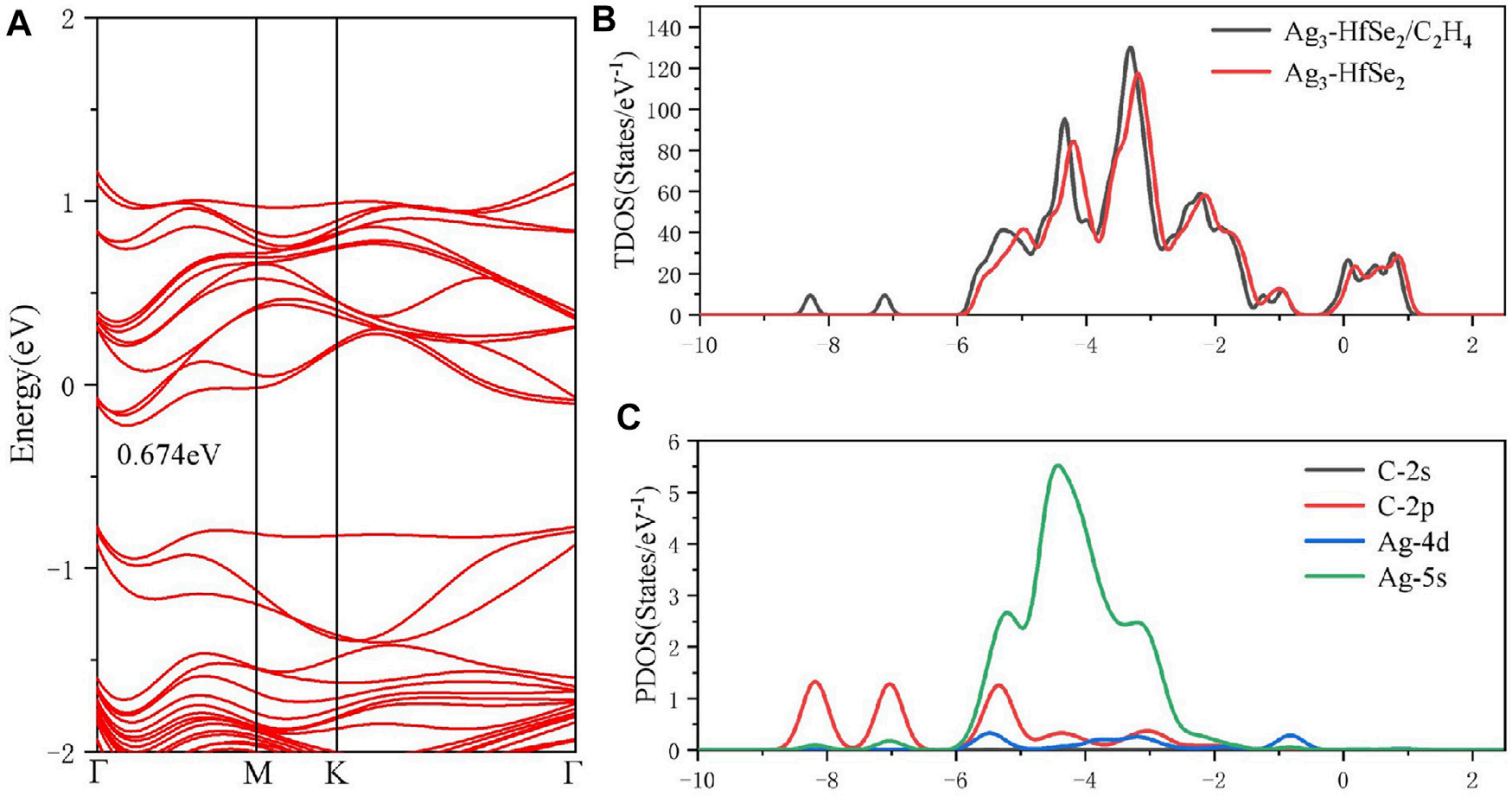
**(A)** Band structure, **(B)** TDOS and **(C)** PDOS of C_2_H_4_ molecules’ adsorption on the Ag_3_-doped HfSe_2_ monolayer.

To better investigate the property of the adsorption of the C_2_H_4_ molecule upon the doped monolayer, three possible adsorption sites and directions were considered. They are as follows, approaching the Ag_3_ cluster in a parallel way and in a vertical way but in two different directions, respectively. Compared with the results of the adsorption energy for different sites, it is the parallel way that holds the lowest value, which is calculated as −0.911 eV. After adsorption, it can be seen that the gas molecule is arrested by the dopant Ag_3_ cluster. The detailed adsorption performance may be depicted, as in Figure 4C, in which the distance between the C atom and Ag atom is measured as 2.400 Å. It confirms that the interaction exists between the C and Ag atoms, and the structure has an obvious change. From the DCD in Figure 4D, it can be observed that the C atoms are surrounded by red areas, and the H atoms are surrounded by blue areas. It means that the H and Ag atoms act as electron donators (Wang et al., 2020). As is shown in Figure 6A, the band structure reveals that the bandgap of the adsorption system is 0.674 eV, which has an increase of 0.156 eV. Compared with the band structure before the adsorption, it can be found that it becomes denser and adds more impurity tracks. It widens the impurity band of the adsorption system, makes the transfer of electrons more conducive, and presents a better adsorption effect (Wu et al., 2017). The result obtained from the TDOS in Figure 6B demonstrates that there is a slight right shift, which is coincident with the analysis of the bandgap mentioned earlier. As demonstrated in Figure 6C, the PDOS reveals that there is a hybridization between the C-2p orbital and Ag-5s and Ag-4d orbitals at −6 ∼ −2 eV. On the basis of the analysis of Mulliken atomic charges, there is 0.221 e transferred from the C_2_H_4_ molecule to the doped system.

Moreover, according to the adsorption of the two gases, it can be concluded that the doped Ag_3_–HfSe_2_ monolayer is more selective for C_2_H_4_ gas molecules. Compared to the electronic properties of the monolayer after the adsorption of CO and C_2_H_4_, it can be seen that the latter holds lower adsorption energy, a wider bandgap, and a higher charge transfer capacity. All the properties mentioned previously contribute to its better performance of adsorption. As a consequence, from the aspect of electron property, it is the C_2_H_4_ system that holds the stronger interaction compared with the CO system. This also indicates its strong potential for applying gas sensing for detecting C_2_H_4_ gas molecules (Chen et al., 2020).

### Analysis of the Frontier Molecular Orbital Theory

**FIGURE 7:**
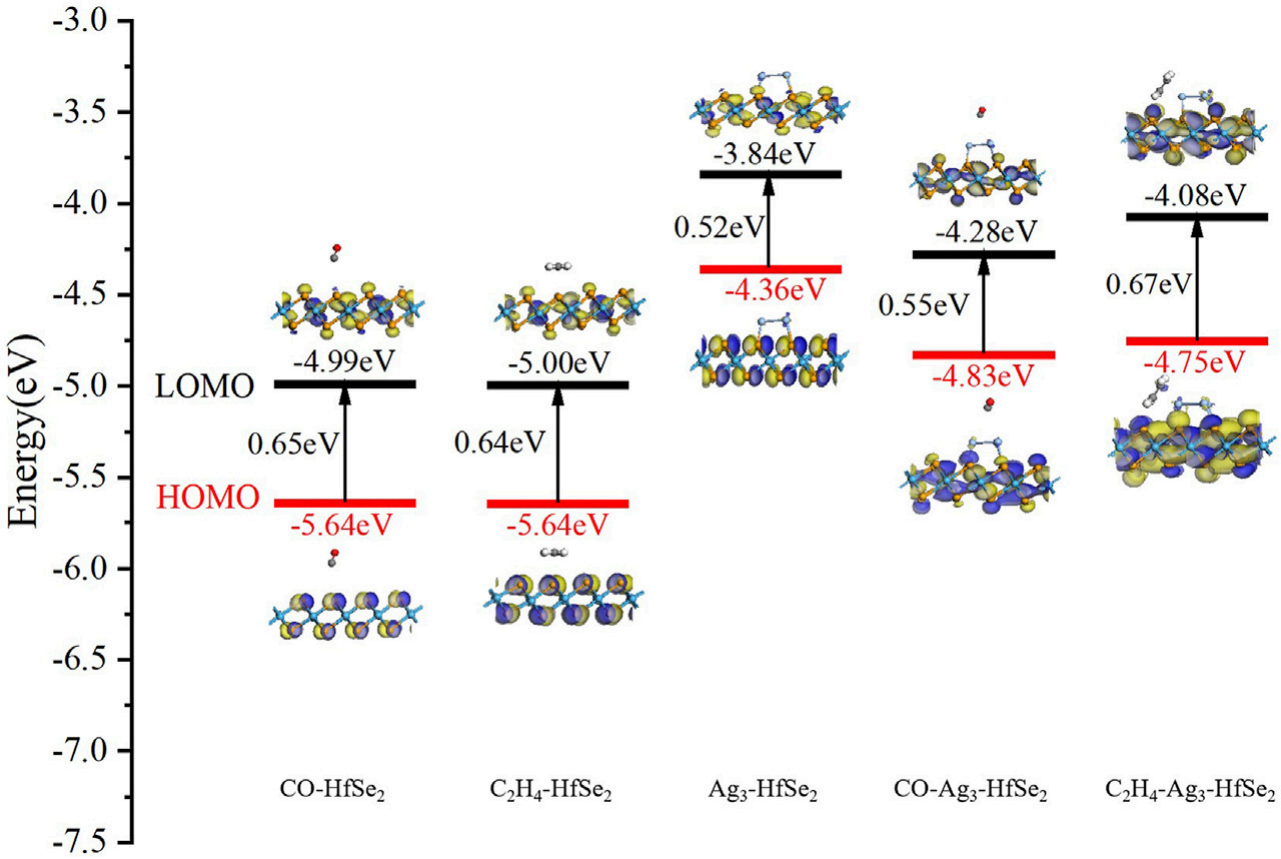
Frontier molecular orbital distributions of CO and C_2_H_4_ adsorption on pristine and Ag_3_–HfSe_2_ monolayers, respectively.

On the basis of the electronic mechanism, the dopant changes the electrostatic potential on the HfSe_2_ monolayer because of its different electron affinities. It contributes to a change in the height of the surface potential barrier or a corresponding change in the resistance value of the semiconductor. Gas molecules are trapped on the monolayer by the Ag_3_ cluster, and the dopant could be defined as a catalyst during the adsorption process (Asif et al., 2019b). It means the dopant can enhance the adsorption nature of gas molecules and accelerate the sensing electron exchange. Based on the frontier molecular orbital theory, the reasons that affect the conductivity change can be reacted directly (Liao et al., 2021). As is known, the resistive gas sensor demonstrates the effect of adsorption on different gas molecules by detecting the resistance change of the material (Asif et al., 2018). As is depicted in Figure 7, it shows the highest occupied orbital (HOMO) and the lowest free orbital (LOMO) before and after the adsorption of C_2_H_4_ and CO molecules on pristine and doped HfSe_2_. Compared with the pristine substrate material, the difference value between the LOMO and HOMO has a slight increase, which is 0.11 and 0.10 eV, respectively. Figure 7 shows the frontier molecular orbital of the doped system, in which the corresponding bandgap between the LOMO and HOMO is 0.52 eV. Moreover, the LOMO and HOMO orbitals are mainly distributed on the side of the doped sites of the Ag_3_ cluster. However, the distribution of the LOMO and HOMO is stretched to the C_2_H_4_ and CO molecules obviously after the interaction between Ag_3_–HfSe_2_ and adsorbed gas molecules. During the adsorption process of the doped system, there have been incremental changes to various extents, which are 0.15 and 0.03 eV for C_2_H_4_ and CO systems, respectively.

In accordance with the molecular orbital theory, the decrease in the bandgap means that the charge transfer of the system is harder. In other words, at a constant temperature, the wider the forbidden band, the harder it is for the electrons to be transferred from the valence band to the conduction band. As a result, from a macro point of view, it is demonstrated as an increase in the resistance but a decrease in the conductivity (Wu et al., 2022). The relation between resistivity and bandgap can be described as follows.

$$\sigma \propto e^{-E_{g}/2K_{B}T}.$$ (3-1)

Here, σ and $E_{g}$ are the conductivity and bandgap of the sensing material, and T and $K_{B}$ mean the Boltzmann constant and the temperature, respectively.

## Analysis of Recovery Characteristics and Electrical Sensitivity

Last but not the least, it is the recovery properties that must be considered for the sensing materials, in which the recovery time is an important reference. Recovery time, also regarded as desorption time, refers to the desorption rate of the adsorbed gas molecules on a gas sensor. Generally speaking, the faster the recovery time, the better is the performance of the gas sensor. The desorption time is defined as formula (4-1).

$$Τ\;=\;A^{-1}\;e^{\frac{E_{ad}}{K_{B}T}}.$$ (4-1)

Here, τ and A represent the desorption time and apparent frequency factor 10^12^ s^−1^, and $E_{ad}$ and T mean the absolute value of the adsorption energy and different test temperatures, respectively. In this article, the test temperatures were defined as 289 K, 358 K, and 418 K, respectively, and the Boltzmann constant is 8.62 × 10^−5^ eV/K (Gui et al., 2020). According to the equation given previously, the desorption time of the adsorbed gas C_2_H_4_ and CO under different test temperatures is demonstrated in Table 3. It can be seen that the desorption time reduces gradually with the increase in the test temperature. In other words, to a certain degree, the higher the ambient temperature of the gas sensing elements, the faster the gas detaches from the gas sensing material. The results calculated are consistent with the previous analysis, including the physisorption of CO and the chemisorption of C_2_H_4_ on the Ag_3_–HfSe_2_ monolayer.

**TABLE 3:** Desorption time (s) of the adsorbed gas under different test temperatures.

Temperature(K)	CO–HfSe_2_	CO–Ag_3_–HfSe_2_	C_2_H_4_–HfSe_2_	C_2_H_4_–Ag_3_–HfSe_2_
298	4.79 × 10^−10^	3.59 × 10^−6^	6.47 × 10^−7^	2,603.7408
358	1.70 × 10^−10^	2.86 × 10^−7^	6.87 × 10^−8^	6.7917
418	8.15 × 10^−11^	4.71 × 10^−8^	1.39 × 10^−8^	0.09774

In addition to the desorption time analyzed previously, it is the electrical sensitivity (E_S_) that must be considered, which is defined in the following equation.

$$E_{S}\;=\left(\sigma_{gas}^{-1}-\sigma_{Ag_{3}-HfSe_{2}}^{-1}\right)/\sigma_{Ag_{3}-HfSe_{2}}^{-1}\;,$$ (4-2)

**FIGURE 8:**
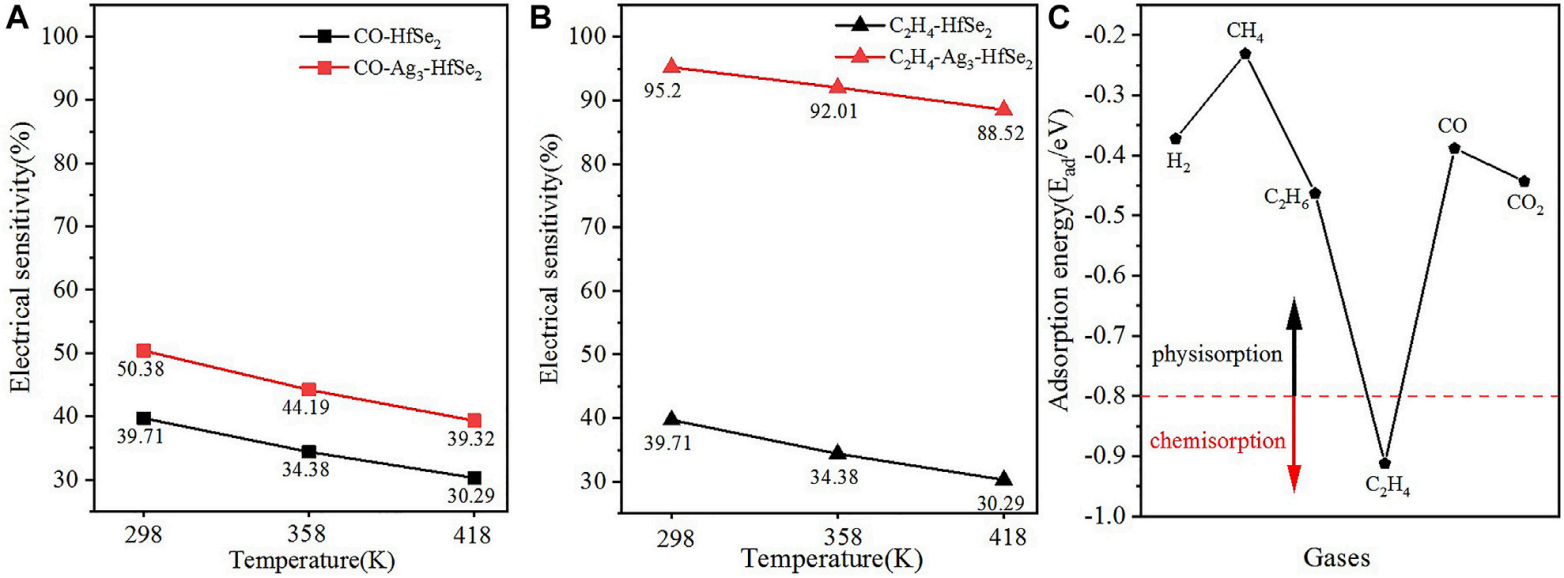
Electrical sensitivity of **(A)** CO and **(B)**C_2_H_4_ on pristine and doped adsorption systems. **(C)** Comparison of the adsorption energy of different gases.

Here, $\sigma_{gas}^{-1}$ and $\sigma_{Ag_{3}-HfSe_{2}}^{-1}$ represent the electrical conductivity of the Ag_3_–HfSe_2_ monolayer before and after adsorption, respectively. The electrical conductivity could be calculated as in Eq. 4–2. The results of the electrical sensitivity are drawn in Figure 8, from which can be seen that after the doping of the Ag_3_ cluster, there are obvious promotions for the electrical sensitivity of two gases. In particular, for the adsorption of C_2_H_4_, the electrical sensitivity changes from 39.71 to 95.2%, revealing its good performance of adsorption (Aziz et al., 2022).

To elucidate the anti-interference for the detection of the C_2_H_4_ molecule, the adsorption energy of different dissolved gases in oil was compared. The gases are H_2_, CH_4_, C_2_H_6_, C_2_H_4_, CO, and CO_2_ respectively. As is shown in Figure 8C, it can be seen that C_2_H_4_ is below the boundary at −0.8 eV, while other gases are above the boundary. In other words, only the adsorption of C_2_H_4_ is chemisorption while others are defined as physisorption. As a consequence, it can be concluded that the detection of C_2_H_4_ based on the Ag3–HfSe_2_ monolayer is of great anti-interference (Aziz et al., 2019).

## Conclusion

In this article, the adsorption properties of the transformer oil decomposition gases (CO and C_2_H_4_) upon Ag_3_–HfSe_2_ are investigated theoretically on the basis of the first principle. The electronic behavior of Ag_3_–HfSe_2_ before and after the adsorption is analyzed and discussed by the band structure, DOS, DCD, and the Mulliken analysis. However, the desorption time is calculated through the adsorption energy, and the electrical sensitivity is analyzed through the bandgap and the frontier molecular orbital theory (Das and Shoji, 2011). After analyzing the adsorption behaviors and the response mechanisms of CO and C_2_H_4_, the main conclusions obtained are as follows. For the doping of the Ag_3_ cluster, the simulation results show that the Ag_3_ cluster is more inclined to be doped at the site T_se1_. There are different adsorption interactions between the gases CO, C_2_H_4_, and the Ag_3_–HfSe_2_ monolayer, including the strong chemical adsorption of C_2_H_4_ and the weak physisorption of CO, respectively. In the adsorption process, C_2_H_4_ was attracted to transfer electrons to the monolayer of Ag_3_–HfSe_2_, whereas CO was just the reverse. After adsorption, the increase in the bandgap contributes to the increase in resistivity of Ag_3_–HfSe_2_, which corresponds to a decrease in conductivity. The resistivity relationship corresponding to the two adsorption systems is as follows: C_2_H_4_ > CO. After the doping of the Ag3 cluster, the electrical sensitivity has a great change for both the gases, including the change from 39.71 to 50.78% for CO and the change from 39.71 to 95.2% for C_2_H_4,_ respectively. Speaking of the anti-interference of the detection for C_2_H_4_, the adsorption energy of different gases was compared. The results illustrate that the detection for C_2_H_4_ based on Ag3–HfSe_2_ is of great anti-interference. Our investigation highlights the high selectivity and better adsorption performance of C_2_H_4_ on the Ag_3_–HfSe_2_ monolayer. It provides guidance for expanding the application range of HfSe_2_ to the sensing materials and analyzes its feasibility to detect transformer oil decomposition theoretically.

## Data Availability

The original contributions presented in the study are included in the article/supplementary material; further inquiries can be directed to the corresponding authors.
